# Bump time-frequency toolbox: a toolbox for time-frequency oscillatory bursts extraction in electrophysiological signals

**DOI:** 10.1186/1471-2202-10-46

**Published:** 2009-05-12

**Authors:** François B Vialatte, Jordi Solé-Casals, Justin Dauwels, Monique Maurice, Andrzej Cichocki

**Affiliations:** 1 Riken BSI, Lab. ABSP, Wako-Shi, Japan; 2 University of Vic, Vic, Spain; 3 MIT, Cambridge, MA, USA

## Abstract

**Background:**

oscillatory activity, which can be separated in background and oscillatory burst pattern activities, is supposed to be representative of local synchronies of neural assemblies. Oscillatory burst events should consequently play a specific functional role, distinct from background EEG activity – especially for cognitive tasks (*e.g*. working memory tasks), binding mechanisms and perceptual dynamics (*e.g*. visual binding), or in clinical contexts (*e.g*. effects of brain disorders). However extracting oscillatory events in single trials, with a reliable and consistent method, is not a simple task.

**Results:**

in this work we propose a user-friendly stand-alone toolbox, which models in a reasonable time a bump time-frequency model from the wavelet representations of a set of signals. The software is provided with a Matlab toolbox which can compute wavelet representations before calling automatically the stand-alone application.

**Conclusion:**

The tool is publicly available as a freeware at the address:

## Background

The structural organization (which elements are relevant) and associated functional role (how these elements play a role in brain dynamics) of electroencephalographic (EEG) oscillations are still far from being completely understood. Oscillatory activity can be separated in background (or ongoing) and transient burst pattern activities. The background EEG is constituted by regular waves, whereas bursts are transient and with higher amplitudes: cortical dynamics do not follow continuous patterns, but instead operates in steps, or frames [1]. These bursts are organized local activities, most likely to be representative of local synchronies. Synchrony among oscillating neural assemblies is a plausible candidate to mediate functional connectivity, and therefore to allow the formation of spatiotemporal representations [2,3]. Oscillatory bursts should consequently play a specific functional role, distinct from ongoing background EEG activity (which does not mean however that background activity cannot convey information). They are correlated with reciprocal dynamic connections of neural structures, which can be considered as distributed local networks of neurons [4]. Together, distant neural assemblies are involved in collective dynamics of synchronous neuronal oscillations [3], taking the shape of oscillatory patterns.

EEG activities are usually analyzed using either time or frequency representations of event related potentials (ERP), which can be interpreted as the reorganization of the spontaneous brain oscillations in response to the stimulus [5,6]. ERP can be further seprated into two sub-groups: event related synchronization (ERS), and similarly event related desynchronization (ERD). The simplest hypothesis concerning the origins of ERP would be additive: following the stimulus onset, in each trial, a transient change of amplitude is observed in a given frequency range, independent of the ongoing signal (the signal is synchronized or dissynchronized in all trials). However, other competing theories could also explain the ERP patterns. The stimulus could induce a change in the phase of ongoing oscillations, without power changes. If the phases of all trials were aligned or dialigned, then after averaging ongoing oscillations an ERS or ERD pattern would arise: this is the so-called theory of phase resetting (*e.g*. [7,8]; see also [9]). Another recent theory explains the generation of event related potentials as a consequence of a baseline shift of ongoing activity [10]. Confronting these three competing theories with experimental facts seems necessary in order to understand the basis of neural dynamics.

It should be noted however that all the above ERP theories are interested in the study of averages of electrophysiological signals in the time domain. ERP were observed in several studies to have visible outcomes even in single trials [11,12], especially observable when using wavelets [6,12,13] which represent the signal with optimal time-frequency resolutions. Local oscillatory events are present in single trials, appearing as transient oscillatory synchronizations (TOS) or transient oscillatory desynchronizations (TOD), corresponding to the presence or absence of a coherent neural assembly [6]. If the additive theory was right, ERS and ERD could be assumed to be the outcome of an average of TOS and TOD events respectively – if not (especially in the phase shift theory), they would have an independent meaning, and it would be even more worthwile to study them. We are not interested here in an averaged outcome (we do not want to study ERP, but TOS and TOD), because the brain itself usually processes information in single trials.

**Figure 1:**
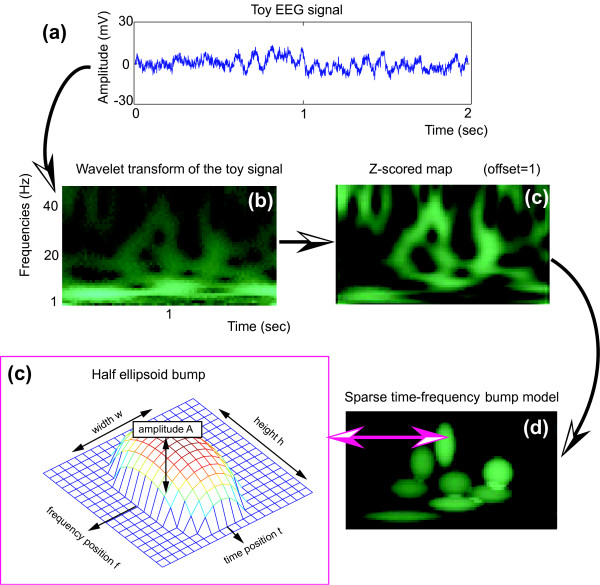
**Sparse time-frequency bump modeling of a toy EEG signal**. (a) The toy EEG signal (Biosemi system, 2048 Hz sampling rate, 2 sec), recorded in rest condition with eyes closed, is first (b) transformed using complex Morlet wavelets, then (c) the map is z-scored (offset = 1). Sparse time-frequency bump modeling decomposes the z-scored map into a sum (d) of half ellipsoid (c) parametric functions (windows of 4 cycles, pruned to the 8 first bumps).

Instead of using average approaches, one could try to analyze directly single trials in the time-frequency plane. However, in the case of time-frequency planes, hundreds of thousands of coefficients are used to represent a signal; and when a large set of signals is to be compared, the complexity of simple graphical matching methods becomes intractable. Analyzing directly this large amount of information leads to complex computations, and either approximate or over-fitted models (this problem is usually termed as the "curse of dimensionality"). We instead advocate a sparsification approach. The main purpose of sparsification approaches is to extract relevant information within redundant data. Sparse time-frequency bump modeling, a 2D extension of the 1D bump modeling described in [14], was developed for this purpose: sparse time-frequency bump modeling extracts the most prominent bursts within a normalized time-frequency map, by modeling them into a sum of parametric functions (see Fig. 1). Bump modeling is however not the only possible sparsification approach. The ridges [15,16] of wavelet maps can be extracted; but while they are sparse, their biological interpretation is not trivial. Multiway analysis [17] allows the simultaneous extraction of multi-dimensional modes, and can be applied to time-frequency representations of electrophysiological signals (e.g. [18]); however it does not allow the independent analysis of transient oscillations. Wavelet packets [16,19] allow the computation of very sparse time-frequency representation (which can be efficient for signal compression, sometimes also for feature extraction); however, because they provide inaccurate time-frequency locations of oscillatory contents, the use of discrete wavelets is not appropriate for signal analysis – especially for electrophysiological data. Finally, the closest method to bump modeling would be matching pursuit [20], which associates a library of functions to a signal – the signal is thereby decomposed into a set of atomic functions, each with specific time-frequency properties (one function could be used to represent a transient oscillation). Other more straightforward attempts were also made at extracting specific atoms of EEG oscillations, like for instance the extraction of narrow band alpha peak epochs [9], or the analysis of a specific time-frequency area (e.g. [21]), but with limitations due to an a priori defined time-frequency area or frequency range [22]. As a comparison, sparse bump modeling allows an automatic broadband modeling of time-frequency atoms, each of them interpreted as transient local activities of neural assemblies (TOS or TOD).

Sparse time-frequency bump modeling was first applied to model invasive EEG (local field potentials), recorded from rats olfactory bulb during a go-no go olfactory memory task [22,23]. Afterwards, it was used to investigate several aspects of brain oscillatory dynamics: scalp EEG data from patients with early stage of Alzheimer's disease (AD) was also successfully analyzed and classified using bump modeling [22,24,25] with a high accuracy (80–93% leave-one-out validation rate). The model was also applied to represent simultaneously time-frequency and space information using a sonification approach [26]. Time-frequency space information was then exploited using a synchrony model: oscillatory burst extracted with bump modeling were used to determine large-scale synchrony – the stochastic event synchrony (SES, [27,28]). Applied to EEG recorded from early AD patients, SES extracted significant differences with age-matched control subjects. These differences were complementary when compared against 30 other synchrony measures [29,30] (uncorrelated with all other 30 measures). Finally, oscillations of steady state visual event potential epochs were extracted (hundred single trial EEG signals) using sparse bump modeling, and an increase of large-scale synchrony measured using SES [31]. This confirms that sparse bump modeling has a wide range of possible applications, from feature extraction to signal modeling and analysis.

However, due to its complexity, it was until now difficult for researchers to reproduce our results: only one external group [32] tried to reproduce results obtained with bump modeling on Alzheimer's disease modeling (they obtained an 80% classification rate). Therefore we present now a toolbox [see Additional file 1] for sparse bump modeling. The software extracts transient oscillatory events (TOS or TOD). We will first describe the method procedure, then demonstrate the toolbox functions with a toy signal. In appendix, we present some details of the improved adaptation and matching methods implemented in the toolbox.

## Implementation

### Method Procedure

ButIf toolbox follows four steps (rationales for this procedure, proofs and technical details are explained in [22], see also Fig. 1): (in Matlab) the signal is first wavelet transformed into time-frequency, then the time-frequency map is z-score normalized; (in the stand-alone software) the map is described by a set of time-frequency window, then parametric functions are adapted within these windows, in decreasing ordered of energy.

The first steps are executed with Matlab, while the last are executed with a stand-alone software. Note however that the stand-alone software can be directly called by Matlab: the whole package can be run from Matlab.

#### Wavelets

Wavelets (see [16,19] for details), especially complex Morlet wavelets [33], have already been widely used for time-frequency analysis of EEG signals [13,34-39]. Complex Morlet wavelets ϑ of Gaussian shape in time (deviation *σ*) are defined as:

(1)

where *σ *and *f *are interdependent parameters, the constraint 2*πft *> 5. The wavelet family defined by 2*πft *= 7, as described in [34], is adapted to the investigation of EEG signals. This wavelet has positive and negative values resembling those of an EEG, but also a symmetric Gaussian shape both in the time and frequency domains – *i.e*. this wavelet locates accurately time-frequency oscillations both in the time and frequency domain.

We scale complex Morlet wavelet ϑ to compute time-frequency wavelet representations of the signal **X **of length *T*:

(2)

where *s*, the scaling factor, controls the central frequency *f *of the mother wavelet. The modulus of this time-scale representation can therefore be used as a positive time-frequency spectrogram, which we will note **C**_**x**_, a time-frequency matrix of dimension *T *× *F*, where *F *scales are used to compute appropriate frequency steps (usually linear or logarithmic, in the case of bump modeling we use linear steps).

#### Z-score

The time-frequency spectrogram is normalized depending on a reference signal. The reference signal is used to determine the usual distribution of the time-frequency map: bump modeling will extract activities with transient amplitudes above or below this usual distribution (*i.e*. high or low z-score). The reference signal **R **of dimension *U *is wavelet transformed into a spectrogram **C**_**r **_of dimension *U *× *F*. The average **M**_**f**_(**C**_**r**_) = [*μ*_1_(**C**_**r**_), *μ*_2_(**C**_**r**_),..., *μ*_*F*_(**C**_**r**_)] and standard deviations **S**_**f**_(**C**_**r**_) = [*σ*_1_(**C**_**r**_), *σ*_2_(**C**_**r**_),..*σ*_*F*_(**C**_**r**_)] are computed from **C**_**r **_for each of the *F *frequencies of the matrix **C**_**r**_. For instance, at frequency *i*:

(3)

and

(4)

The z-scored map **Z**_**x **_is obtained through normalization of the wavelet map **C**_**x **_using these values:

(5)

In event related studies, the reference signal is usually a signal recorded during a rest period, just before the stimulus period (the so-called pre-stimulus period). In rest condition, the reference signal can be either the signal itself (self-reference signal); or a statistic derived from a group of signals (as used e.g. in [26]). The minimal number of samples of the reference signal should be determined with regards to the lowest frequency range (because low frequencies have longer cycles than high frequencies), so that a sufficient number of cycles is present – otherwise, the reference statistic would not be significant.

#### Windowing

The z-scored map is analyzed in a time-frequency area of interest defined by the user, with lowest frequency *f*_*m *_and highest frequency *f*_*x*_. The z-scored map **Z**_**x **_is described by a set of windows *ω*(*f*, *t*) with *f *∈ [*f*_*m*_, *f*_*m *_+ 1,...*f*_*x*_], *t *∈ [*b*_*t*_, *b*_*t *_+ 1...*T *- *b*_*t*_] the position on the general time-frequency map respectively in frequency and time. Each *ω *has its own dimensions *H *× *W *(height and width), determined depending on the time-frequency resolution at the window's central frequency. The dimension *W *is determined to have a duration of a fixed (usually 4) number of cycles (for instance, *W *= 1 sec. for activities with central frequency at 4 Hz, *W *= 100 msec. for activities at 40 Hz). The dimension *H *is determined as the ratio of *W *to the time-frequency resolution (see [22]). The limit *b*_*t *_is determined so that *b*_*t *_= *W*/2 for the windows at the frequency *f*_*m *_(low frequency windows are larger than high frequency windows). In other words, the left and right limits of the modeled area are vertical, and constrained by the lower frequency so as to allow the modelisation of bumps centered at these limits [22] (similarly, the z-score map is modelled with extract upper and lower borders in frequencies).

#### Parametric functions

We use half ellipsoid functions (see Fig. 1) to model the normalized time-frequency map. The half ellipsoid boundaries are defined as:

(6)

where *y *and *x *are respectively the time and frequency position of the adaptation window on the time-frequency map (fixed parameters), *f *and *t *are respectively the time and frequency position of the bump on the time-frequency map, *h *and *w *are respectively the height and width of the bump, and *A *is its amplitude. All points lying inside these boundaries are non zero; outward points have null values. This is expressed by:

(7)

where *λ *is a small positive value (*λ *is non zero for computational reasons:  could not be computed for infinitesimal values of Ψ in C++).

Hence the adaptation error to be minimized will be:

(8)

Adaptation is performed using a combination of first and second order gradient descent (using the BFGS algorithm [40]). More details on the adaptation procedure can be found in [41] and in the appendix.

#### C++ implementation

The stand-alone software was implemented in C++ for better speed, and higher stability when manipulating large data sets (Bump modeling usually computes hundreds of Frobenius norms in the time-frequency plane, it can be time-consuming – our previous implementation using Matlab was about 100 times slower).

Exchange files between Matlab and the C++ software (the '.wvf' and '.bdc' files) store data as integers. Float or double precision real numbers are recoded into integers (this can be seen in the script used to save and load these files), because Matlab and C++ Builder appear to use differing symbolic representations. The wavelet maps are implemented as objects, with time-frequency contents partitioned into windows using a chain list object. Each window is sorted using an energy function, based on the matching procedure described in the appendix. The modeling procedure selects iteratively the best window, performs the parameter adaptation, re-computes the energy of neighboring windows, and finally calls a method performing a fast sorting of the windows. In the end, this procedure avoids redundant computations: the set of window energy is computed once and for all at the beginning of the modeling. The adaptation itself is applied to bump functions, implemented as virtual methods. The algorithm uses the step gradient described in the appendix, the BFGS algorithm part was re-coded from a *Numerical Recipes *original code [40]).

### ButIf Toolbox

**Figure 2:**
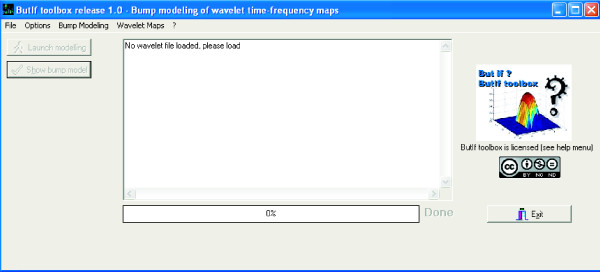
**ButIf toolbox main window**. Screen capture of the stand-alone software main window. The software opens wavelet files (.wvf) generated with Matlab, and extracts a sparse half ellipsoid bump model.

The toolbox is separated in two parts: One is a stand-alone software (Fig. 2) developed using Borland C++ Builder 2006. This standalone software is used to model bumps from wavelet maps. It needs a Windows XP environment to run. The second one is a set of Matlab m-files that perform the wavelet transform and call the software. The resulting bump model can be displayed using a Matlab function ('display_bumps.m'). The toolbox uses several parameters, they are detailed both in the Matlab files (Matlab_'.m' files, such as 'demo_basic.m') as comments, and on the website [42]. Most parameters are related to the wavelet transform itself (minimal frequency, maximal frequency, etc..., documentation can be found in [42]). Let us explain here those related with bump modeling: z-score offset, window dimension, and convergence/pruning criterion.

#### z-score offset

The first step of bump modeling is wavelet time-frequency representation. Afterwards, the z-score normalization is applied to the map. We will refer to the positive z-score values as transient oscillatory synchronization (TOS) components: if the signal is recorded during a stimulation, these oscillatory peaks are likely to be constituent of ERS (assuming the additive theory is valid), and probably carry specific information related to the stimulus (as they are representative of local neural assembly synchronizations). If the signal is recorded in pre-stimulus period or in rest condition, these oscillations are representative of organized oscillatory bursts. Z-score returns values in ℝ, but bump modeling only accepts values in ℝ^+ ^as inputs. In order to model TOS, we reject the negative components of the map with a threshold, the z-score offset *ϕ*. The thresholded map  is obtained with:

(9)

Usual values of *ϕ *are in the [0–3] range, it corresponds to the proportion of ongoing activity which will be rejected (a z-score of 2 corresponds to 95%, a z-score of 3 to 99%). For very clean signals, a low z-score is possible, for noisy signals, the threshold should be higher. The parameter 'offset_val' represents *ϕ*.

We will refer to the negative z-score values as transient oscillatory desynchronization (TOD) components: if the signal is recorded during a stimulation, these oscillatory peaks are likely to be constituent of ERD (assuming the additive theory is valid), and probably carry specific information related to the stimulus(as they are representative of local neural assembly desynchronizations). If the signal is recorded in pre-stimulus period or in rest condition, these oscillations are representative of unusually disorganized oscillatory bursts. These negative z-score values in ℝ^- ^can be extracted into a threshold map :

(10)

In order to model , the parameter 'offset_val' must be given the value -1 in the model header.

#### Window Dimension

**Figure 3:**
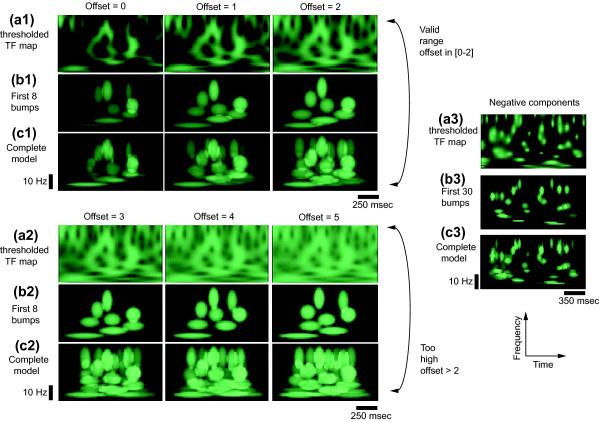
**Effect of the z-score offset**. Wavelet thresholded z-score map  (a1, a2, a3), and bump model for offsets *ϕ *∈ [-1..5] (b1, b2, b3, c1, c2, c3). Left: wavelet negative z-score maps  (a1, a2) were first modeled until eight bumps were obtained (b1, b2), then continued until completion (c1, c2). If the z-score offset is too high (a2), the map is not well fitted (c1 *vs*. c2). Right: wavelet negative z-score map  (a3), and its bump model (b3, c3). The modeling was performed first until 30 bumps were obtained (b3), then continued until completion (c3). Negative z-score events are shorter than positive ones (a1, a2 *vs*. a3), which leads to the modeling of more events (b1, b2 *vs*. b3).

Wavelets have a specific time-frequency resolution depending on their central frequency [16,19]. This resolution corresponds to their precision in time and frequency. Consequently, the representation of a high frequency transient activity will be narrow in time and spread in frequency – conversely for low frequency transient activities. This is taken into account when establishing the adaptation windows. The dimension *H *× *W *(height and width) of the windows *ω *depends on the time-frequency resolution at the window's central frequency: the dimension *W *is determined to have a duration of a fixed number of time periods, and the dimension *H *is determined as the ratio of *W *to the time-frequency resolution (see above, and [22]). This dimension should always be determined to fit the average size of transient synchrony events, but is adaptive with frequency (it does not usually need to be changed for analyzing low or high frequency signals – because it is expressed in cycles). The parameter 'header.cote' in the toolbox represents this fixed number, *i.e*. the number of oscillation one wishes to model. It was observed that TOS last around three to four cycles (oscillate three to four times then disappear) [22,36]. Negative events (TOD) are shorter in duration, lasting approximately two time periods. The parameter 'header.cote' should usually be four to model TOS events; and two to model TOD events (as an illustration, see Fig. 3). However, one can change these parameters if he wishes to study its relevance. In this case, take into consideration that windows encompassing too many cycles would attempt to model several events with one bump; whereas a too short window would split the map in many unncessary atoms.

#### Convergence and Pruning

We first design a model with the largest number of bumps – within a reasonable computation time. To that effect, the fraction of the total intensity of the map modeled by a given bump is computed:

(11)

This value is compared with a threshold *F*_*t*_: when three consecutive bumps have *F *<*F*_*t*_, modeling stops. The parameter 'header.limit' (usually = 0.2) represents this threshold.

A tradeoff must be performed between accuracy and relevance (also termed 'bias-variance dilemma'): if the number of bumps in the model is too low, the latter will not be accurate; if it is too large, irrelevant non-organized information from the background EEG will be modeled. When modeling is finished using the above termination criterion, we use a pruning strategy. The Matlab program 'prune_model.m' can be used to perform this pruning. Pruning can be performed with four options: pruning to remove only abnormal bumps (bumps with abnormally small amplitude, width or height, *i.e*. below 5.10^-2^); pruning to remove bumps with a threshold *F*_*t*2 _≥ *F*_*t*_; pruning to remove all bumps after the *N *first modeled (modeling order); or pruning to remove all bumps after the *N *first in time order. Users can combine these options, in order to obtain a clean and accurate representation.

## Results

Here we will demonstrate how this toolbox works using a toy signal, and illustrate the use of the three main parameters (z-score offset, window dimension, and convergence/pruning). All files (signal, Matlab demonstration '.m' files, application) can be downloaded on the bump toolbox project website. The toy signal ('sig_example.mat', see Fig. 1) is an EEG signal taken from one channel recorded in rest eyes closed condition (Biosemi system, 2048 Hz sampling rate, 2 sec). This demonstration can be reproduced by launching demo_zscore under Matlab 7.0 (with the wavelet toolbox). Note that due to border effects of the wavelet transform, 500 msec are automatically rejected on both sides of the wavelet map by the toolbox prior to bump modeling – hence only one second was analyzed in the following examples.

### z-score offset

We vary the parameter 'offset_val' in the [0–5] range to show its effect on modeling (offset_val > 3 is usually not worth try, it is only shown for demonstrative purpose). The resulting models (Fig. 3, left, a1-2, b1-2, c1-2) are in the folder 'result demo_zscore). Obviously, a too permissive z-score offset (here offset_val > 2) will introduce strong bias in the model.

Using the parameter offset_val = -1, the negative components  can be modeled (Fig. 3, right, a3, b3, c3). As illustrated on the figure, the negative events are even more transient, and needs usually more bumps to be properly represented.

### Window Dimension

**Figure 4:**
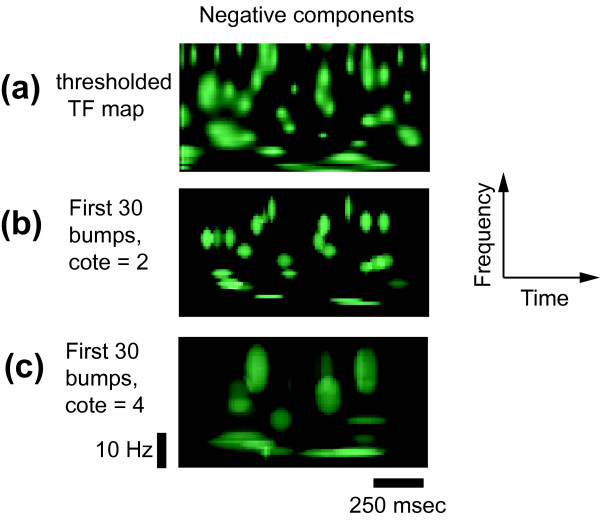
**Effect of windows dimensions**. Example of effect of the windows dimension parameter. The negative z-score  (a) of the toy signal is modeled. Here, the correct windows dimension (b), with header.cote = 2 cycles, is compared with a too large windows dimension parameter (c), with header.cote = 4 cycles. Using inappropriate constraints leads to unprecise modeling.

We introduced above the usual limits for the window dimension. We will illustrate here the effect of this parameter. We again modeled the negative components ; but this time instead of using the adequate window dimension parameter (header.cote = 2) we used too large windows (header.cote = 4). In this condition, the minimized error *E *will be lower when two or more events are modeled by one bump only (which is not desirable, see Fig. 4).

### Convergence and Pruning

The results of demo_zscore are shown with (Fig. 3, b1-3) or without pruning (Fig. 3, c1-3). The first model is performed with a limit *F*_*t *_= 0.2 without pruning, the second model is pruned with a fixed number of bumps (options 1 and 3 in 'prune_model.m').

## Conclusion

The ButIf toolbox allows the extraction of time-frequency oscillatory events. Whatever theory (additivity, phase resetting or baseline shift) would best explain ERP, extracting transient oscillations in single trials is always relevant: if ERP are due to a change of amplitude, more transient oscillations should be observed. If instead a phase shift of the signal is observed, then the transient oscillations position will change both for transient oscillations an background EEG; combined with changes in the number of these oscillations in case we admit the theory of baseline shifts. Contrary to averaging studies, using sparse time-frequency bump modeling, analyzing the amplitude, number, or alignment of TOS and TOD becomes feasible in single trials. In other words, whatever the generation hypothesis, bump modeling allows a better understanding of the mechanism behind the presence of an ERP, because it facilitates the independent analysis of transient oscillations in single trial, which provides complementary information. The idea is not to neglect the information of ongoing activity, but instead to allow a separate study of transient oscillations on one hand, and ongoing activity on the other hand. In the end, even the presence or absence of ERP is not relevant, as the analysis of transient synchronizations and desynchronizations can be relevant even in rest conditions (e.g.[29], [24]). We stress again that bump modeling is a method well suited in order to study the characteristics of transient oscillations, and should not be confused with ERP averaging.

This approach has already provided new insights in EEG and LFP signals ([22,28]), and will hopefully allow significant progress in the investigation of brain dynamics. Nevertheless, although this toolbox is user-friendly and reasonably efficient, it is very dependent upon parameter settings. More specifically, one parameter is critical when the toolbox is used for applications such as SES [27,28], classification [22], or statistical analysis [31]: the pruning threshold. A visual inspection (using the script 'display_bumps.m') will allow users to refine this parameter. Automatic approaches to determine the best parameter are also possible. Such an optimization is usually performed with an *ad-hoc *algorithm depending on the application: for instance, if the application is a classification, this parameter has to be estimated during the validation procedure. As another example, in [22], we described an algorithm which can be used to optimize this threshold when event-related paradigms are used.

The software transforms a set of EEG signals first into a set of wavelet transforms (sored in Matlab '.mat' files), then into a bump model (more details are provided in the manual 'BUTIF Toolbox FAQ.pdf'). The outcome is a set of parameters (corresponding to the parametric bump functions) stored in structured variable inside a Matlab '.mat' file. This variable can be visualized (displaying a bump spectrogram, using the script 'display_bumps.m') or used for statistics. This provides a better understanding of the structure of EEG oscillations (TOS and TOD). Despite this toolbox provides many tools, some maintenance work will remain necessary in the future to improve and upgrade it. For instance, the choice of atom functions affects the modeling. We originally chose half ellipsoid for their sparsity (only five parameters); but despite a dissymmetric 2D curve would have more parameters, it might better represent the dissymetry of wavelet representations (larger for lower frequencies). Until now we obtained good results with half ellipsoids, but this does not mean that we might not find a better solution. Using other curves is on the list of our future experimentations and developments for the toolbox. Additionally, the pruning criterion, as explained above, is critical. It would certainly benefit from further investigations. New developments, however, will not be possible without interactions with users (we invite questions, suggestions and comments; please see the website and its discussion forum for more details). Hence, the current version only lays the foundation stone of a long-term project.

## Availability and requirements

**Project name**: ButIf toolbox

**Project home page**: [42]

**Operating system(s)**: Windows XP

**Programming language**: Matlab/C++ Builder 2006

**Other requirements**: Matlab 6.0 or 7.0 with wavelet toolbox is necessary to run this package (the wavelet toolbox could also be replaced by a freeware wavelet toolbox, such as the Uvi_Wave toolbox of Universidad de Vigo in Spain, which contains the complex Morlet wavelet).

**License**: Creative Commons License (CC-by-nc-nd) – Anyone can use this software for academic applications providing they properly reference our work when publishing research results obtained with this toolbox (citing the present paper, and [22]).

**Any restrictions to use by non-academics**: the software is restricted to non commercial applications.

## Authors' contributions

FBV developed the concept of time-frequency bump modeling during his Ph.D., created the software, wrote the first draft of the paper, and created the first version of the website. JS-C helped in debugging of the final version of the software, and for the preparation of the website (especially the FAQ section). Reviewed the draft of the paper. JD helped in the theoretical development of bump modeling. Reviewed the draft of the paper. MM helped in the design of the website, was involved in the preparation of all illustrations. AC helped in the theoretical developments of bump modeling. Reviewed the draft of the paper. All authors read and approved the final manuscript.

## Appendix

### Appendix A: Improved Adaptation

In the previous implementation of bump modeling [22], we optimized all the bump function parameters (*A*, *h*, *w*, *f*, and *t*) simultaneously (first using iterations of first order gradient descent, followed by iterations of the BFGS [40] algorithm). An improved adaptation can be obtained by optimizing the parameters stepwise, with a priority depending on the order of their derivatives. When comparing of the parameters derivatives (*dE/dA*, *dE/dh*, *dE/dw*, *dE/df *and *dE/dt*), we observe that the term -2 is common to all these derivatives, multiplyed by a term in ℝ+: they will all have the same sign [41]. The slope of the adaptation will then be dependant on the multiplicands *m *applied to -2*E*: *m *= 1 for *dE/dA*; *m *is a positive value in [0 - *A*] in numerator divided by a variable of order 3 for *dE/dh *and *dE/dw*; *m *is a positive value in [0 - *A*] in numerator divided by a variable of order 2 for *dE/df *and *dE/dt*.

This would probably be working correctly with properly normalized parameters, however we are here adapting parameters of different ranges: *A *∈ [0 - 1], while *h *and *f *∈ [1 - *H*/2] and *w *and *t *∈ [1 - *W*/2] with *H *and *W *usually >> 1. Therefore, the multiplicands corresponding to the three above case will be of the order *m *∈ *O*(1) in case (1), and *m *∈ *O*(*x*^-3^) in case (2) and *m *∈ *O*(*x*^-2^) in case (3). Practically speaking, it means that the parameters adaptation should be performed stepwise (first *h *and *w*, then *f *and *t*, and finally *A*). We improved the quality and speed of convergence by performing the following stepwise estimation of these parameters:

1. Update *h *and *w *until both their derivatives are below a threshold *t*_Ψ_.

2. Update *f *and *t *until both their derivative are below a threshold *t*_*pos*_. If at anytime *dE/dh *or *dE/dw *becomes above *t*_Ψ_, go back to 1.

3. Update only *A*, until its derivative is below a threshold *t*_*A*_. If at anytime *dE/dh *or *dE/dw *becomes above *t*_Ψ_, go back to 1. If at anytime *dE/df *or *dE/dt *becomes above *t*_*pos*_, go back to 2.

The adaptation is still performed using the BFGS [40] algorithm.

### Appendix B: Improved window matching

In the previous version of bump modeling [22], the best candidate window **Ω **was selected as:

(12)

Because these windows are used to determine the initial condition of the function adaptation, finding the best suitable window is primordial. The new optimized method is more related to matching pursuit [20], in that we will match the window content **w**(*f*, *t*) with a prototype bump function *ξ*_**w**(*f*, *t*) _= *ξ*(*A*_*w*_, *h*_*w*_, *l*_*w*_, *f*_*w*_, *t*_*w*_, *y*, *x*) with *f*_*w *_= *h*_*w *_= *H*/2, *x*_*w*_, and *A*_*w *_the highest peak in the window:

(13)

For each window **w**(*f*, *t*), we compute the corresponding matrix **Ξ**_*w*_(*f*, *t*) of values of this prototype function. The best window is thus matched as:

(14)

Where: denotes the Frobenius inner product, and ||·||_*F *_indicates the Frobenius norm. This generalize the matching criterion of matching pursuit methods to 2D data, with the difference that for bump modeling, high z-score has priority on best fit. Therefore, contrary to matching pursuit, the product is not normalized by the norm of **w**.

## Supplementary Material

Additional file 1 **All_In_One.zip**. This file contains the ButIf toolbox 1.0, including the stand-alone software, Matlab package, demo files, sample data, and results of the demo. The 'documents' subfolder contains a manual (BUTIF Toolbox FAQ.pdf). Click here for file

## References

[B1] Freeman W (2006). A cinematographic hypothesis of cortical dynamics in perception. International Journal of Psychophysiology.

[B2] Le Van Quyen M (2003). Disentangling the dynamic core: a research program for a neurodynamics at the large-scale. Biological Research.

[B3] Cosmelli D, Lachaux J, Thompson E, Zelazo P, Moscovitch M, Thompson E (2007). Neurodynamical approaches to consciousness. The Cambridge Handbook of Consciousness.

[B4] Varela F, Lachaux J, Rodriguez E, Martinerie J (2001). The Brainweb: Phase Synchronization and Large-Scale Integration. Nature Reviews Neuroscience.

[B5] Başar E (1980). EEG-brain dynamics: Relation between EEG and brain evoked potentials.

[B6] Başar E, Demilrap T, Schürmann M, Başar-Eroglu C, Ademoglu A (1999). Oscillatory brain dynamics, wavelet analysis, ands cognition. Brain and Language.

[B7] Moratti S, Clementz B, Gao Y, Ortiz T, Keil A (2007). Neural mechanisms of evoked oscillations: Stability and interaction with transient events. Human Brain Mapping.

[B8] Klimesch W, Sauseng P, Hanslmayr S, Gruber W, Freunberger R (2007). Event-related phase reorganization may explain evoked neural dynamics. Neuroscience and biobehavioral reviews.

[B9] Risner M, Aura C, Black J, Gawne T (2009). The Visual Evoked Potential is independent of surface alpha rhythm phase. Neuroimage.

[B10] Nikulin V, Linkenkaer-Hansen K, Nolte G, Lemm S, Müller K, Ilmoniemi R, Curio G (2007). A novel mechanism for evoked responses in the human brain. European Journal of Neuroscience.

[B11] Effern A, Lehnertz K, Schreiber T, David P, Elger C (2000). Nonlinear denoising of transient signals with application to event related potentials. Physica D.

[B12] Quiroga R, Sakowitz O, Başar E, Schürmann M (2001). Wavelet transform in the analysis of the frequency composition of evoked potentials. Brain Research Protocols.

[B13] Vialatte F, Solé-Casals J, Cichocki A (2008). EEG windowed statistical wavelet scoring for evaluation and discrimination of muscular artifacts. Physiological Measurements.

[B14] R D, Maison-Blanche P, Quenet B, Dreyfus G (2007). Automatic ECG wave extraction in long-term recordings using Gaussian mesa function models and nonlinear probability estimators. Comput Methods Programs Biomed.

[B15] Delprat N, Escudié B, Guillemain P, Kronland-Martinet R, P T, B T (1992). Asymptotic wavelet and Gabor analysis: Extraction of instantaneous frequencies. IEEE Trans Inform Theory.

[B16] Mallat S (1999). A wavelet tour of signal processing.

[B17] Carroll J, Chang J (1970). Analysis of individual differences in multidimensional scaling via an n-way generalization of 'Eckart-Young' decomposition. Psychometrika.

[B18] Acar E, Aykut-Bingol C, Bingol H, Bro R, Yener B (2007). Multiway analysis of epilepsy tensors. Bioinformatics.

[B19] Percival D, Walden A (2000). Wavelet Methods for Time Series Analysis.

[B20] Mallat S, Z Z (1993). Matching Pursuits with Time-Frequency Dictionaries. IEEE Transactions on Signal Processing.

[B21] Gruber T, Müller M (2005). Oscillatory brain activity dissociates between associative stimulus content in a repetition priming task in the human EEG. Cerebral Cortex.

[B22] Vialatte F, Martin C, Dubois R, Haddad J, Quenet B, Gervais R, G D (2007). A Machine Learning Approach to the Analysis of Time-Frequency Maps, and Its Application to Neural Dynamics. Neural Networks.

[B23] Vialatte F, Martin C, Ravel N, Quenet B, Dreyfus G, Gervais R (2003). Oscillatory activity, behaviour and memory, new approaches for LFP signal analysis. Proceedings of the 35th annual general meeting of the European Brain and Behaviour Neuroscience Society (EBBS'03): 17–20 September Barcelona, Spain -Acta Neurobiologiae Experimentalis.

[B24] Vialatte F, Cichocki A, Dreyfus G, Musha T, Shishkin SL, Gervais R (2005). Early Detection of Alzheimer's Disease by Blind Source Separation, Time Frequency Representation, and Bump Modeling of EEG Signals (invited presentation). LNCS 3696, Proceedings of the International Conference on Artificial Neural Networks 2005 (ICANN'05): 11–15 September 2005, Warsaw, Poland.

[B25] Vialatte F, Cichocki A, Dreyfus G, Musha T, Rutkowski T, Gervais R (2005). Blind source separation and sparse bump modelling of time frequency representation of EEG signals: New tools for early detection of Alzheimer's disease. Proceedings of the IEEE Workshop on Machine Learning for Signal Processing 2005 (MLSP'05): 28–30 September 2005, Mystic CT, USA.

[B26] Vialatte F, Cichocki A (2006). Sparse Bump Sonification: a New Tool for Multichannel EEG Diagnosis of Mental Disorders; Application to the Detection of the Early Stage of Alzheimer's Disease. LNCS 4234, Proceedings of the 13th International Conference on Neural Information Processing (ICONIP'06): 3–6 October 2006, Hong Kong, China.

[B27] Dauwels J, Vialatte F, Cichocki A (2007). A novel measure for synchrony and its application to neural signals. Proceedings of the 32nd IEEE International Conference on Acoustics, Speech, and Signal Processing (ICASSP'07): 15–20 April 2007, Honolulu, USA.

[B28] Dauwels J, Vialatte F, Weber T, Cichocki A (2009). Quantifying statistical interdependance by message passing on graphs: algorithms and applications to neural signals – Part A. Neural Computation.

[B29] Dauwels J, Vialatte F, Cichocki A (2008). On Synchrony Measures for the Detection of Alzheimer's Disease based on EEG. LNCS 4984, Proceedings of the 14th International Conference on Neural Information Processing (ICONIP'07): 13–16 November 2007; Kita Kyushu, Japan.

[B30] Dauwels J, Vialatte F, Rutkowski T, Cichocki A, Platt J, Koller D, Singer Y, Roweis S (2008). Measuring Neural Synchrony by Message Passing. Advances in Neural Information Processing Systems 20.

[B31] Vialatte F, Dauwels J, Rutkowski TM, Cichocki A, Wang R, Gu F, Shen E (2008). Oscillatory Event Synchrony During Steady State Visual Evoked Potentials. Advances in Cognitive Neurodynamics, Proceedings of the International Conference on Cognitive Neurodynamics 2007.

[B32] Buscema M, Rossini P, Babiloni C, Grossi E (2007). The IFAST model, a novel parallel nonlinear EEG analysis technique, distinguishes mild cognitive impairment and Alzheimer's disease patients with high degree of accuracy. Artificial Intelligence In Medicine.

[B33] Kronland-Martinet R, Morlet J, Grossmann A (1988). Analysis of sound patterns through wavelet transforms. International Journal on Pattern Recognition and Artificial Intelligence.

[B34] Tallon-Baudry C, Bertrand O, Delpuech C, Pernier J (1996). Stimulus specificity of phase-locked and non-phase-locked 40 Hz visual responses in human. Journal of Neuroscience.

[B35] Düzel E, Habib R, Schott B, Schoenfeld A, Lobaugh N, McIntosh AR, Scholz M, Heinze HJ (2003). A multivariate, spatiotemporal analysis of electromagnetic time-frequency data of recognition memory. Neuroimage.

[B36] Caplan J, Madsen J, Raghavachari S, Kahana M (2001). Distinct patterns of brain oscillations underlie two basic parameters of human maze learning. J Neurophysiol.

[B37] Li X, Yao X, Fox J, Jefferys J (2007). Interaction dynamics of neuronal oscillations analysed using wavelet transforms. Journal of Neuroscience Methods.

[B38] Slobounov S, Hallett M, Cao C, Newell K (2008). Modulation of cortical activity as a result of voluntary postural sway direction: An EEG study. Neuroscience Letters.

[B39] Vialatte F, Bakardjian H, Prasad R, Cichocki A (2009). EEG paroxysmal gamma waves during Bhramari Pranayama: A yoga breathing technique. Consciousness and Cognition.

[B40] Press WH, Flannery BP, Teukolsky SA, T VW (2002). Numerical recipes in C: The art of scientific computing.

[B41] Vialatte F, Dauwels J, Solé-Casals J, Maurice M, Cichocki A (2009). Improved Sparse Bump Modeling for Electrophysiological Data. Proceedings of the 15th International Conference on Neural Information Processing (ICONIP'08) – Part I, LNCS.

[B42] Vialatte F, Solé-Casals J, Dauwels J, Maurice M, Cichocki A ButIf toolbox project. http://www.bsp.brain.riken.jp/bumptoolbox/toolbox_home.html.

