# Supplementary material for: Bump time-frequency toolbox: a toolbox for time-frequency oscillatory bursts extraction in electrophysiological signals
Source: BMC Neurosci. 2009 May 12;10:46. doi: 10.1186/1471-2202-10-46 (PMC2690596; doi:10.1186/1471-2202-10-46)
Supplement: Additional file 1 — All_In_One.zip. This file contains the ButIf toolbox 1.0, including the stand-alone software, Matlab package, demo files, sample data, and results of the demo. The 'documents' subfolder contains a manual (BUTIF Toolbox FAQ.pdf). [file 1471-2202-10-46-S1.zip › All_In_One/documents/BUTIF Toolbox FAQ.pdf]

BUTIF Toolbox FAQ

GENERAL INFORMATION 2

INSTALLATION 4

GETTING STARTED 5

MODELING PARAMETERS 9

THE MODEL 10

General Information

What is BUTIFtoolbox made for?
Why do we need to extract oscillatory bursts?
What is a time-frequency map?
What is a bump?

What is BUTIFtoolbox made for?

BUTIFtoolbox is used to extract transient oscillatory dynamics from signals. Until now, it was succesfully applied to LFP (Local Field Potentials) and EEG (Electroencephalographic) signals. Applications to other fields could be researched, as for instance in speech processing.

Why do we need to extract oscillatory bursts?

The structural organization and associated functional role of electroencephalographic (LFP or EEG) oscillations are still far from being completely understood. Oscillatory activity can be separated in background and burst pattern activities. The background EEG is constituted by regular waves, whereas bursts are transient and with higher amplitudes. These bursts are organized local activities, most likely to be representative of local synchronies. They should consequently play a specific functional role, distinct from background electroencephalographic activity.

What is a time-frequency map?

A time-frequency map conveniently represents simultaneously time and frequency information. The Fourier spectrum of a signal represents the frequency content of the signal, the signal itself is in the time domain. In time-frequency maps, the frequency spectum is given for each time step so that one can see the evolution of the frequencies. The best time-frequency resolution is achieved when time-frequency maps are computed using wavelets.

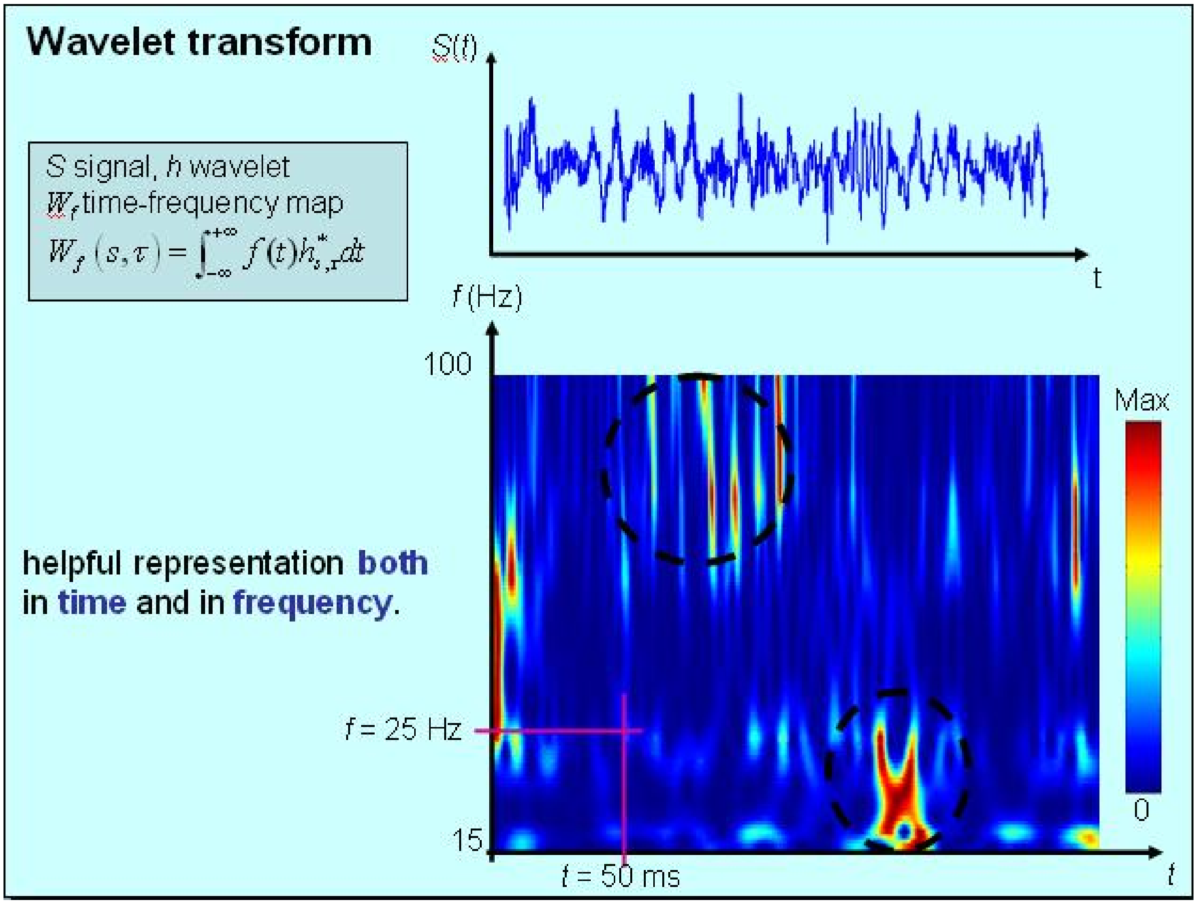

What is a bump?

In our context, we loosely define a bump as a parametric function, which is used for atomic decomposition of the time-frequency map. Usually, half-ellipsoid bumps are used. See Vialatte et al. 2007.

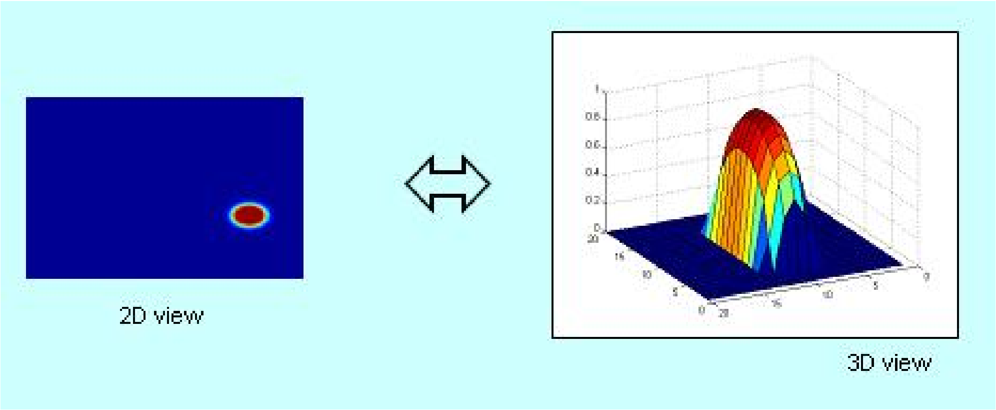

Installation

Downloading the toolbox
Installing the packages

Downloading the toolbox

Go to the [Download] section. The "Application" section contains the core files of the toolbox. The "Demo" section contains sample m-files used to run the toolbox. For first time users, dowload the all-in-one package and install everything. Click on the links and save the file on your local hard disk.

Installing the packages

To install the toolbox, you need first to unzip the packages. The m-files must be defined in matlab's path. The ".exe" file must be placed in Matlab's working directory (= the current directory of Matlab), because matlab cannot find executable files in its path.

The ".exe" software is used to open ".wvf" files and created ".bdc" files, while the m-file package computes these ".wvf" (wavelet time-frequency maps) files and opens the ".bdc" files obtained when bumps are modeled. You should the try to launch the demos to check if the toolbox works properly (see the section getting started).

Getting started

how must I proceed to do an experiment with the toolbox?
how can I use my own signals with the toolbox?
what demo_basic do? how can I see the results?
what demo_multi do? how can I see the results?
what demo_toolbox do? how can I see the results?
what demo_zscore do? how can I see the results?

how must I proceed to do an experiment with the toolbox?

First of all you need to have the toolbox properly installed. Afterwards you should get familiar with the basics about time-frequency bump modelling. You then finally need in addition a signal, stored in Matlab as a vector.

First off all, the toolbox will transform the signal into a wavelet time-frequency map. This time-frequency map will be saved on you disk in a '.mat' file. However, the ButIf.exe stand-alone software for bump modeling cannot read Matlab files. This is why a transfer file '.wvf' is also saved (you can delete his file once modeling is done). Similarly, after bump modeling the file is saved as a '.bdc' (also a transfer file that can be deleted once modeling is over). In the end, a '.mat' file that contains a varialbe 'model' is produced - this is the bump model.

The modeling is performed following these steps:

- calling butif_toolbox.m or creating a script from demo_basic to open the signal (see section how can I use my own signals with the toolbox?)
- the wavelet files (.mat and .wvf) are created
- the bump modeling software (ButIf.exe) is called, opens the '.wvf' file and saves a '.bdc' file
- the toolbox opens the '.bdc' and the '.mat' wavelet file, and creates the '.mat' bump model from them.

how can I use my own signals with the toolbox?

There are two solutions:

1) calling butif_toolbox(signal, sampling_rate) will compute the bump model of 'signal'. You can see the parameters needed by typing help butif_toolbox in Matlab interface:

butif_toolbox(signal, samplingrate, name, reference, freqmin, freqmax, freqstep, downsample, offset_val, limit, maxi)
or more usually:
butif_toolbox(signal, samplingrate, name);

Calling this file will compute a basic sparse time-frequency representation from the input vector "signal" (dimension 1xN or Nx1).
samplingrate = samplingrate of the input signal in Hz.
only "signal" and "samplingrate" are necessary inputs, and [] can be given as parameter to skip an input variable: for instance demo_vectorial(rand(1000,1),100,[],[],[],20,[],1) is accepted.

- name = name of output files (.mat, .wvf, .bdc). If omitted, the outputfile will be 'default';
- reference = a reference signal used to compute the zscore. If this input is omitted, signal will be self-referenced
- freqmin = minimal frequency for modelling. If freqmin is omitted, the minimal frequency will be determined as the lowest possible frequency according to signal duration (limit of wavelet lateral border effects, with min = 1Hz, so that at least 80% of the signal is modelled).
- freqmax = maximal frequency for modelling. If freqmax is omitted, the maximal frequency will be determined as 1/5th of the sampling rate (with max=85Hz).
- freqstep = the step between frequencies. If the step is omitted, freqstep=1 will be used
- downsample = downsampling of the wavelet map before bump modelling (considerably accelerates modeling). If downsample is not precised, it will be assigned the value of the Nyquist rate (1/2 of sampling rate).
- offset_val = the z-score offset (value in [1-3]; or -1 for desynchronizations). If omitted, offset=+1 will be used.
- limit = modeling limit, usually in [0.1-0.3] (in percentage of the total energy). If omitted, limit = 0.2 will be used.
- maxi = maximal number of bump modelled (usually in [100-500]). If omitted, maxi = 300 will be used.

2) Editing a script (changing demo_basic).

The 'demo_basic.m' file can be easily modified to be used as a script file to open your own signals. The loaded signal has to be replaced with the user's one; and the parameters have to be checked to correspond to the new signal.

what demo_basic do? how can I see the results?

demo_basic models the toy EEG signal sig_example.mat. After computing the wavelet transform, it computes its bump model.

The resulting files can be found in the working directory (or current directory):
- a file 'wave_demobasic.mat' containing the wavelet transform (Matlab compatible),
- a file 'default.wvf' that can be opened with ButIf.exe,
- a file 'default.bdc' containing the resulting bump model (tranfer file)
- the bump model translated into Matlab data file 'default.mat'.

In order to see the result, it is only needed to open this file (default.mat). The contained variable 'model' is the resulting bump model. In the folder 'result_demobasic' you can find the expected results, that you can compare with yours to check if the toolbox is working properly.

what demo_multi do? how can I see the results?

demo_basic models the sample EEG recording SSVEP_5Hz_1trial.mat. After computing the wavelet transform, it computes the bump model of all channels.

The resulting files can be found in the working directory (or current directory):

- a file 'wave_demomulti.mat' containing the wavelet transform (Matlab compatible)
- a file 'demomulti.wvf' that can be opened with ButIf.exe
- a file 'demomulti.bdc' containing the resulting bump model (tranfer file)
- the bump model translated into Matlab data file 'demomulti.mat'.

In order to see the result, it is only needed to open this file (demomulti.mat). The contained variable 'model' is the resulting bump model. In the folder 'result_demomulti' you can find the expected results, that you can compare with yours to check if the toolbox is working properly.

what demo_toolbox do? how can I see the results?

'demo_toolbox.m' is an example file. It simply calls the butif_toolbox Matlab function in order to model the toy EEG signal sig_example.mat.

The resulting files can be found the the working directory (or current directory):

- a file 'wave_default.mat' containing the wavelet transform (Matlab compatible)
- a file 'default.wvf' that can be opened with ButIf.exe
- a file 'default.bdc' containing the resulting bump model (tranfer file)
- the bump model translated into Matlab data file 'default.mat'.

In order to see the result, it is only needed to open this file (default.mat). The contained variable 'model' is the resulting bump model. In the folder 'result_demotoolbox' you can find the expected results, that you can compare with yours to check if the toolbox is working properly.

what demo_zscore do? how can I see the results?

demo_basic models several times the toy EEG signal sig_example.mat, each time with a different z-score: -1,0,1,2,3,4 and 5. After iteratively computing the wavelet transform, it computes its bump model.

The resulting files can be found in the working directory (or current directory):

- a file 'wave_demozscore.mat' containing the wavelet transform (Matlab compatible)
- a file 'demozscore.wvf' that can be opened with ButIf.exe
- a file 'demozscore.bdc' containing the resulting bump model (tranfer file)
- the bump model translated into Matlab data file 'demozscore.mat'.

In order to see the result, it is only needed to open this file (demozscore.mat). The contained variable 'model' is the resulting bump model. In the folder 'result_demozscore' you can find the expected results, that you can compare with yours to check if the toolbox is working properly.

Modeling parameters

[zscore offset](#)
[frequency step](#)
[sampling rate and downsampling](#)

zscore offset

z-score is computed using a reference signal, it is a statistical balancing of the wavelet map, usually necessary before bump modeling. The z-score offset is used to remove a portion of the time-frequency map background activity:

- if offset is in the [0-3] range, low energy activity is removed. For instance, offset = 2 removes all activity falling below 95% of the usual signal energy distribution at each frequency, while offset = 3 removes 99% ("usual" energy distribution being estimated from the reference period).
- if offset = -1, the positive peaks of the maps will be removed, while the negative peaks are modeled (the absolute value of the negative z-score is used for modeling). These acitvities reflects oscillatory patterns of abnormally low energy as compared to the reference signal.

frequency step

The modeling is performed by linear frequency steps. The default value is 1, which means that integer frequencies are modelled (e.g. 1,2,3,4,5,6..,50).

When low-frequency activity is investigated, the toolbox will provide more efficient modelling if a smaller step is used (e.g. 0.25 for the delta 1-4 Hz or theta 4-8 hz ranges in EEG).

sampling rate and downsampling

Despite nyquist rate is half of the higher frequency, it is generally advisory to use a sampling rate of 5 times the higher modeled frequency. This is what the toolbox does by default when the time-frequency map is computed. Once the map is computed, this constraint is relaxed, and the map can be downsampled to the nyquist rate limit (i.e. two times the higher investigated frequency).

The model

[where is the bump model?](#)
[what are the different fields of the bump model](#)
[what are the important parameters of the model?](#)
[how can I visualize the resulting model?](#)
[why are the leftmost and rightmost components not modeled?](#)
[how can I export the important parameters of the model?](#)

where is the bump model?

Alter the modeling ende, the bump model is saved in Matlab's work directory, in a ".mat" file, under the name which was given as a parameter (the field "name" when "butif(name)" was called). This file can be oponed with Matlab, and contains a variable "model". This variable is the bump model itself.

what are the different fields of the bump model?

In a bump model file, you will invariably find a 'model' variable. The field cell_dec contains the model. The 'model' variables uses the following structure:

version: Version of the file (ButIf toolbox 1.0 uses wvf/bdc files version 3)
cote: Window size used for modeling
freqmin: minimal frequency modeled
freqmax: maximal frequency modeled
freqsmp: step between each frequencies (usually = 1)
freqdown: sampling rate after downsampling of the wavelet map
ByUp: frequency boundary at the highest frequency
(bump center goes up to the highest frequency, which means that there is a need for a little more information above freqmax - see Vialatte et al. 2007 [[science direct link](#)])
ByDn: frequency boundary at the lower frequency
(bump center goes up to the highest frequency, which means that there is a need for a little more information below freqmin - see Vialatte et al. 2007 [[science direct link](#)])
Bx: time boundary at the right and left of the wavelet map
(bump center goes until the left and right limits of the map, which means that there is a need for a little more information at the left and right of the map - see Vialatte et al. 2007 [[science direct link](#)])
resols: time-frequency resolution windows used for modeling (in pixels)
*dec: bump decomposition. All decomposition are concatenated in an [N*num x 5] matrix. Models with less than num bumps are completed with lines of '-1'.*
num: maximal number of bump for all the N signals modeled.
N: number of signals modeled.
restes: measure of the remainders of the wavelet map in %
erreur: evolution of the cost function for each bump

cell_dec: bump decomposition organised in a structure
maxnorm: norm of the wavelet map (value before normalization)
varspec: standard deviation of the wavelet map's amplitudes for each frequency

spectre: average of the wavelet map's amplitudes for each frequency (equivalent to a frequency Spectrum).

what are the important parameters of the model?

The field “model.dec” contains the model, in a matrix representation. The bumps are represented with 5 variables, as vectors of parameters (concatenated as an [N*num x 5] matrix in “model.dec”). The 5 values of these vector correspond to the bump amplitude, width in frequency, width in time, position in frequency, and position in time (A,df,dt,f,t). You can compute the matrix representation of a half ellipsoid function using the script “calc_demi_ellips.m” (which can be visualized using the matlab routine “imagesc”). Models with less than num bumps are completed with lines of '-1'.

The bumps were modeled within time-frequency windows. These windows are in the field “model.windows”, with a similare representation as “model.dec” (N*num x 4] matrix). The 4 parameters of each window corresponds to its extent in frequency and time, and its position in frequency and time (df,dt,f,t).

how can I visualize the resulting model?

Call the script “display_bumps.m”. This script displays, one by one, all the bump models of the variable “model”. The “bumpogram” matrixes can be retrieved in the output variable (a cell representation of each bumpogram matrix, which can be visualized using the matlab routine “imagesc” or 2D representations, or “surf” for 3D representations).

why are the leftmost and rightmost components not modeled?

When modeling a map, we define left and right limits in time for modeling. Bumps are allowed if their centers fall within these limits (this is done when the map is distributed into a set of windows). The corresponding early or late wavelet components, which were not modeled, are outside this limit (they are outside the modeled zone).

A second limitation “effect” is visible on the maps, which is due to the shape of bumps: low frequency bumps have larger extents than high frequency one, so that on the modeled maps some “holes” are visible on top right and left. In other words, despite the limit is vertical, its effect on the image depends on the frequency.

how can I export the important parameters of the model?

The script “get_bump” will automatically retrieve the j-th bump from the i-th model (5x1 vector of parameter, and 4x1 vector of parameters.
